# An analysis of the graph processing landscape

**DOI:** 10.1186/s40537-021-00443-9

**Published:** 2021-04-09

**Authors:** Miguel E. Coimbra, Alexandre P. Francisco, Luís Veiga

**Affiliations:** 1 INESC-ID, R. Alves Redol 9, 1000-029 Lisbon, Portugal; 2 Instituto Superior Técnico, Universidade de Lisboa, Av. Rovisco Pais 1, 1049-001 Lisbon, Portugal

**Keywords:** Graph processing, Distributed systems, Online processing, Graph representation, Dataflow programming

## Abstract

The value of graph-based big data can be unlocked by exploring the topology and metrics of the networks they represent, and the computational approaches to this exploration take on many forms. For the use-case of performing global computations over a graph, it is first ingested into a graph processing system from one of many digital representations. Extracting information from graphs involves processing all their elements globally, which can be done with single-machine systems (with varying approaches to hardware usage), distributed systems (either homogeneous or heterogeneous groups of machines) and systems dedicated to high-performance computing (HPC). For these systems focused on processing the bulk of graph elements, common use-cases consist in executing for example algorithms for vertex ranking or community detection, which produce insights on graph structure and relevance of their elements. Many distributed systems (such as Flink, Spark) and libraries (e.g. Gelly, GraphX) have been built to enable these tasks and improve performance. This is achieved with techniques ranging from classic load balancing (often geared to reduce communication overhead) to exploring trade-offs between delaying computation and relaxing accuracy. In this survey we firstly familiarize the reader with common graph datasets and applications in the world of today. We provide an overview of different aspects of the graph processing landscape and describe classes of systems based on a set of dimensions we describe. The dimensions we detail encompass paradigms to express graph processing, different types of systems to use, coordination and communication models in distributed graph processing, partitioning techniques and different definitions related to the potential for a graph to be updated. This survey is aimed at both the experienced software engineer or researcher as well as the graduate student looking for an understanding of the landscape of solutions (and their limitations) for graph processing.

## Introduction

Graph-based data is found almost everywhere, with examples such as analysing the structure of the World Wide Web [1–3], bio-informatics data representation via *de Bruijn* graphs [4] in metagenomics [5, 6], atoms and covalent relationships in chemistry [7], the structure of distributed computation itself [8], massive parallel learning of tree ensembles [9] and parallel topic models [10]. Academic research centres in collaboration with industry players like Facebook, Microsoft and Google have rolled out their own graph processing systems, contributing to the development of several open-source frameworks [11–14]. They need to deal with huge graphs, such as the case of the Facebook graph with billions of vertices and hundreds of billions of edges [15].

### Domains

We list some of the domains of human activity that are best described by relations between elements—graphs:

#### Social networks

They make up a large portion of social interactions in the Internet. We name some of the best-known ones: Facebook (2.50 billion monthly active users as of December 2019 [16]), Twitter (330 million monthly active users in Q1’19 [17]) and LinkedIn (330 million monthly active users as of December 2019 [18]). In these networks, the vertices represent users and edges are used to represent friendship or follower relationships. Furthermore, they allow the users to send messages to each other. This messaging functionality can be represented with graphs with associated time properties.

#### World Wide Web

Estimates point to the existence of over 1.7 billion websites as of October 2019 [19], with the first one becoming live in 1991, hosted at CERN. Commercial, educational and recreational activities are just some of the many facets of daily life that gave shape to the Internet we know today. With the advent of business models built over the reachability and reputation of websites (e.g. Google, Yahoo and Bing as search engines), the application of graph theory as a tool to study the web structure has matured during the last two decades with techniques to enable the analysis of these massive networks [1, 2].

#### Telecommunications

These networks have been used for decades to enable distant communication between people and their structural properties have been studied using graph-based approaches [20, 21]. The vertices in these networks represent user phones, whose study is relevant for telecommunications companies wishing to assess closeness relationships between subscribers, calculate churn rates, enact more efficient marketing strategies [22] and also to support foreign signals intelligence (SIGINT) activities [23].

#### Recommendation systems

Graph-based approaches to recommendation systems have been heavily explored in the last decades [24–26]. Companies such as Amazon and eBay provide suggestions to users based on user profile similarity in order to increase conversion rates from targeted advertising. The structures underlying this analysis are graph-based [27–29].

#### Transports, smart cities and IoT

Graphs have been used to represent the layout and flow of information in transport networks comprised of people circulating in roads, trains and other means of transport [30–32]. The Internet-of-Things (IoT) will continue to grow as more devices come into play and 5G proliferates. The way IoT devices engage for collaborative purposes and implement security frameworks can be represented as graphs [33].

#### Epidemiology

The analysis of disease propagation and models of transition between states of health, infection, recovery and death are very important for public health and for ensuring standards of practices between countries to protect travellers and countries’ populations [34–37]. These are represented as graphs, which can also be applied to localized health-related topics like reproductive health, sexual networks and the transmission of infections [38, 39]. Real-life epidemics are perhaps at the forefront of examples of this application of graph theory for health preservation, with the most recent example as COVID-19 [40].

**Fig. 1:**
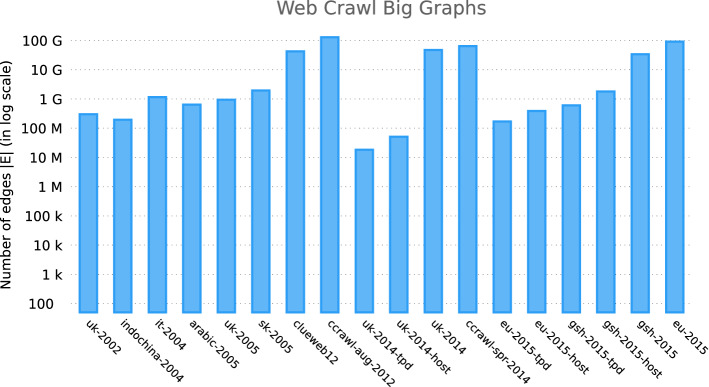
Web graph edge counts for domain crawls since the year 2000 (in log scale)

Other types of data represented as graphs can be found [41]. To illustrate the growing magnitude of graphs, we focus on web graph sizes of different web domains in Fig. 1, where we show the number of edges for web crawl graph datasets made available by the Laboratory of Web Algorithmics [42] and by Web Data Commons [43].

### Motivation

We include this section in this survey to highlight three reasons. Firstly, the recent years have seen a positive tendency in the field of all things related to graph processing. As its aspects are further explored and optimized, with new paradigms proposed, there has been a proliferation of multiple surveys [44–50]. They have made great contributions in systematizing the field of graph processing, by working towards a consensus of terminology and offering discussion on how to present or establish hierarchies of concepts inherent to the field. Effectively, we have seen vast contributions capturing the maturity of different challenges of graph processing and the corresponding responses developed by academia and industry.

The value-proposition of this document is therefore, on a first level, the identification of the dimensions we observe to be relevant with respect to graph processing. This is more complex than, for example, merely listing the types of graph processing system architectures or the types of communication and types of coordination within the class of distributed systems for graph processing. Many of these dimensions, if not all, are interconnected in many ways. As the study of each one is deepened, its individual overlap with the others is eventually noted. For example, using distributed systems, it is necessary to distribute the graph across several machines. This necessity raises the question of how to partition the graph to distribute it. Afterwards, as a natural consequence, it is necessary to define the coordination (e.g. synchronous versus asynchronous) between nodes of the system. Orthogonally to this, the relation between data and computation in graph processing must be defined. The vertices of a graph may represent posts in a social network, while the edges dictate follower relationships. But to the systems that process them, one could specify the units of computation to be solely the vertices, exclusively the edges or components of the graph (and the definition of *component* in this case would be required too). Herein, we first note the cornerstone aspects of the graph processing field from individual works and from existing surveys: dimensions and definitions.

Secondly, we provide an exhaustive list of systems for graph processing, sharing their year of debut and distinctive features. We explore the different types that exist:

#### Single-machine

They may simply require copious amounts of memory, or instead employ compression techniques for graph processing. Orthogonal to this is, and depending on the machine configuration, the processing time for large graphs is also an important challenge. There are techniques that rely on heuristics on the graph to improve execution time, taking advantage of graph structure as well as specific properties of popular algorithms. Examples include increasing the speed of the Louvain community detection method [51] by using heuristics such as informing the vertex community-joining decision process with information above the level of specific vertices, or assigning a colouring scheme so that vertices of the same colour are processed in parallel without adjacent vertices being processed concurrently. Other approaches make use of heuristics such as differentiating processing of vertices based on vertex degree, or to rely on the application of partitioning techniques to enable the processing of graphs which are larger than single-machine memory capacity.

#### Multi-machine

Distributed systems which can be a cluster of machines (either homogeneous or heterogeneous) or special-purpose high-performance computing systems (HPC), requiring coordination at different levels. There are systems in the literature with different design principles which influence the granularity with which parallelism and coordination are performed. General-purpose data processing systems which also have libraries for graph processing do not consider the fine details of graph-structured data, while systems designed with a focus on graph processing enable fine-grained techniques to increase performance.

On the third level, and also considering a scope of meta-analysis, we discuss the structuring of the field that is presented in existing surveys. We complement it with our own highlight of important relations between graph processing concepts and a chronological analysis of the field of graph processing.

##### Document roadmap

This rest of the paper is organized around the following main sections. *Graph Algorithms: Natures and Types* highlights relevant aspects of graph-processing tasks and different types of graph algorithms. *Computational Representations* details important computational representations of graphs which typically use compression techniques. *Graph Processing: Computational Units and Models* analyses how graphs are conceptually manipulated in many contributions of the field’s state of the art, and the different levels of granularity. *Dimension: Partitioning* presents the most-known approaches to decomposing graph-based data into computational units for parallelism and distribution, showcasing models with different levels of granularity. *Dimension: Dynamism* enumerates scenarios with different definitions of *dynamism* in graph processing, from the graph representing temporal data to the manipulated graph representation being constantly updated with new information. *Dimension: Workload* discusses the nature of graph processing workloads and different scopes such as analytics and storage. *Single-Machine and Shared-Memory Parallel Approaches* presents these types of architecture and describes important state-of-the-art marks. *High-Performance Computing* is focused on multi-core and multi-processor architectures. *Distributed Graph Processing Systems* enumerates systems which focus on distributing the graph across machines to enable processing. We finish with *Conclusion* and final remarks.

## Graph algorithms: natures and types

There are several aspects inherent to graph-processing tasks. Graphs have properties which may be extrapolated using specific algorithms, from computing the most important vertices (e.g. using an arbitrary function like PageRank [52]), finding the biggest communities (for which there is a choice of many algorithms) or the most *relevant* paths (for a definition of relevancy). An algorithm that processes the whole graph (as opposed to localized information queries expressed with graph query languages seen previously) is typically executed in parallel fashion when the resources for parallelism are available. When implementing these algorithms, whether the developer manually implements the parallelism or merely uses such a functionality offered by an underlying framework (e.g. Apache Spark [53] or Apache Flink [14]), some challenges must be considered. This means that while the field of graph processing is developed with the goal of improving how we manipulate and extract value from graph-based data, as the techniques to achieve this end become more refined, other aspects of graph structures gain prominence as challenges to them.

We list and comment here the major types of challenges of parallel graph processing identified in a previous study [54]:

1. Data-driven computations: A graph has vertices and edges which establish how computations are performed by algorithms, making graph applications data-driven. We see this observation shift the focus to data—what should the elementary unit of computation be? In this survey we go over multiple solutions in the literature, considering the computation from the perspective of vertices [8, 14, 53, 55], edges [56] and sub-graphs [57].
2. Irregular problems: The distribution of edges and vertices usually does not constitute uniform graphs that form embarrassingly parallel problems, whose benefits from simple parallelism are easier to achieve [58]. We note that after defining the unit of computation in a graph, care needs to be taken when assigning parts of the graph to different processing units. Skewed data will negatively impact load balance [46] unless tailored approaches are undertaken, which take into account different types of graph properties such as scale-free [59] in their designs [60, 61].
3. Poor locality: Locality-based optimizations offered by many processors are hard to apply to the inherently irregular characteristics of graphs due to poor locality during computation. We believe it is important to mention how this manifests when using distributed systems (clusters) to process the graphs. To mitigate this, techniques may be used for example to replicate specific vertices based on properties such as their degree, or to use specific graph partitioning strategies when working with vertex-centric approaches [50].
4. High data-access-to-computation ratio: The authors note that a large portion of graph processing is usually dedicated to data access in graph algorithms and so waiting for memory or disk fetches is the time-most consuming phase relative to the actual computation on a vertex or edge itself. We note one approach [62] to this problem that focused on balancing network and storage latencies with computation time to minimize the impact of underlying data accesses in a cloud computing setting.

### Algorithms

Graph algorithms that execute globally over all elements of a graph have a distinct nature from those solved with graph query languages—the scope of computation is drastically different with respect to the computational resources needed to satisfy it. We note, however, some graph databases such as Neo4j have extensions like the Neo4j APOC Library for languages like Cypher to start algorithms with global computation from the graph query language [63].

A previous survey on the scalability of graph processing frameworks [64, Sec. 3.3] defines a categorization of graph algorithms, which we reproduce here: *Traversals*. Starting from a single node, they employ recursive exploration of the neighbourhood until a termination criteria is met, like reaching a desired node or a certain depth. Instances of this are for example calculating single-source shortest-paths (SSSP), *k*-hop neighbourhood or breadth-first searches (BFS). *Graph analysis*. Algorithms falling into this scope aim at understanding the structural properties and topology of the graph. They may be executed to grasp properties like the diameter (greatest distance between any pair of vertices), density (ratio of the number of edges |*E*| with respect to the maximum possible edges) or degree distribution. *Component identification*. Concept: a *connected component* is a subset of graph vertices for which there is a path between any pair of vertices. Finding connected components is relevant to detect frailties in the networks that graphs represent. The connections between these components are called *bridges*, which if removed, will separate connected components. *Communities detection*. The groups called *communities* consist of sets of vertices that are close to each other within a community than to those outside it. There are different techniques to compute them such as minimal-cut sets and label propagation, among others. *Centrality measures calculation*. These represent the importance of a vertex with respect to the rest of the network. Many definitions exist such as PageRank and betweenness centrality, for example. There also heuristics to measure the relevance of edges such as spanning edge betweenness [65]. *Pattern matching*. Related to algorithms aimed at recognizing or describing patterns, known as *graph matching* in this context. *Graph anonymization*. To produce a graph with similar structure but making it so the entities represented by vertices and edges are not identifiable. Two examples of anonymization procedures are *k*-degree and *k*-neighbourhood anonymity.

Underlying the computations that take place to solve graph-specific tasks lies the granularity. How are the vertices, edges and properties of graphs processed or stored? This required building a bridge from these mathematically-defined elements to the bits and bytes of computers.

## Computational representations

Here we detail terms and concepts which are known in graph theory. We include preliminary notions that serve as a basis to familiarize the reader with the language used in scientific documents on graph applications, processing systems and novel techniques. In the literature [66], a graph *G* is written as $G = (V, E)$—it is usually defined by a set of vertices *V* of size $n = |V|$ and a set of edges *E* of size $m = |E|$. Vertices are sometimes referred to as *nodes* and edges as *links*. An undirected graph *G* is a pair (*V*, *E*) of sets such that $E \subseteq V \times V$ is a set of unordered pairs. If *E* is a set of ordered pairs, then we say that *G* is a directed graph. Between the same two vertices there is usually at most one edge; if there are more, then the graph is called a *multigraph* (note: an ordered graph in which a pair of vertices share two edges in opposite direction is not necessarily a multigraph). Multigraphs are more common when looking at the applications and use-cases for graph databases such as Neo4j [67], where one may model more than one relation type between the same vertices. Additionally, given a graph $G = (V, E)$, the set of vertices of *G* is written as *V*(*G*) and the set of edges as *E*(*G*). More commonly, we write $V(G) = V$ and $E(G) = E$.

Underlying all the ways to extract information from graphs is their digital representation. It is important to understand the set of operations to be performed over the graph and its size in order to guide the choice of representation. To represent the edges, perhaps the two most well-known approaches are the adjacency list and adjacency matrix. The choice of using an adjacency list or a matrix usually depends on the amount of edges in the graph.

**Fig. 2:**
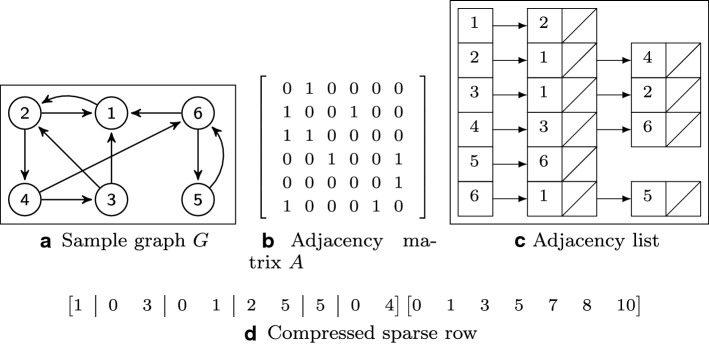
Simple computational representations of sample directed graph *G* shown in **a**

Consider a given graph $G = (V, E)$. If |*E*| is close to the maximum number of edges that a graph can sustain ($|E| \simeq |V|^2$), then it is a *dense* graph and it makes more sense to choose the adjacency matrix (performance-wise). However, if the graph is *sparse* ($|E| \ll |V|^2$), where most nodes are not connected, it can be efficiently represented (storage-wise) with an adjacency list. While the matrix consumes more space than the adjacency list, it allows for constant-time access. We show in Fig. 2 an example of different representations for the same graph.

Figure 2a shows a sample graph *G*, for which the adjacency matrix is shown in Fig. 2b and the corresponding adjacency list in Fig. 2c. The first row of the adjacency matrix *A* represents the outgoing edges of vertex 1, which is connected to vertex 2. It is common in the literature [68, Ch. 22] to use the subscript notation $A_{i,j}$ to refer to the presence of a specific edge in matrix *A* (the notation is relevant for theoretical purposes even if using another type of representation) starting from vertex *i* and targeting vertex *j*:

1 $$\begin{aligned} A_{i,j} = {\left\{ \begin{array}{ll} 1\, \text {if there is an edge from}\ i\ \text {to}\ j,\\ 0\, \text {otherwise.} \end{array}\right. } \end{aligned}$$

Matrix *A* also takes on a particular configuration depending on the graph being directed or undirected. In the later case, there is no explicit sense of source or target of an edge, leading to symmetry in matrix *A*. Implementations of graph processing systems often represent undirected graphs as directed graphs such that the undirected edge between a pair of vertices is represented by two directed edges in opposite directions between the pair.

There are more space-efficient ways to represent a graph, and they become a necessity when exploring the realm of big graphs. The choice between an adjacency list or matrix is bound to the density of the graph. But to justify other representation types, factors such as graph size, storage limitations and performance requirements need special focus. The compressed sparse row (CSR), also known as the *Yale format*, is able to represent a matrix using three arrays: one containing non-zero values; the second containing the extents of rows; the third storing column indices. Figure 2d shows a representation in this format (we omit the array the array containing the non-zero values as they are all one in this case).

Let us consider that indices are zero-based. The array on the left side is the column index array, where the pipe character | separates groups which belong to each row of *A*. The second row (index 1) of *A* has elements at position indexes 0 and 3 in *A*. Therefore, the second group of the column index array has elements [0, 3]. The array on the right is the row index array which has one element per row in matrix *A* and an element which is the count of non-zero elements of *A* at the end of the array (there are variations without this count). For a given row *i*, it encodes the start index of the row group in the column index array (on the left in Fig. 2d). This way, for example, the second row of matrix *A* (Fig. 2b) has row index 1 in *A*. Then, looking at the row index array (the one on the right), as the second row of matrix *A* has row index 1, we access the elements with indices [1, 2] in the row index array, which returns the pair (1, 3), indicating that the second row (index one) of *A* is represented in the column index array starting (inclusive) at index 1 and ending at index 3 (exclusive). If we look at the column index array and check the elements from index 1 (inclusive) to 3 (exclusive), we get the set of values $\{0, 3\}$. And if we look at the second row in *A*, column index 0 and column index 3 are exactly the positions of the edges in *A* for that row. Generally, for a matrix *M*’s row index *i*, we access indices $[i, i+1]$ in the row index array, and the returned pair dictates the starting (inclusive) and ending (exclusive) index interval in the column index array. The set of elements in that interval in the column index array contains the indices of the columns with value 1 for row index *i* in *M*. We point the reader to [69] for details on its representation and construction. There is also the compressed sparse column (CSC), which is similar but focused on the columns, as the name suggests.

Other approaches take advantage of domain-specific properties of graphs. Such is the case of WebGraph [1], which exploits certain properties of web graphs to represent them with increased compression. An important property they exploit is *locality*, as many links stay within the same domain, that is, if the web graph is lexicographically ordered, most links point close by. Another property is *similarity*: pages that are close by in the lexicographical order are likely to have sets of neighbours that are similar. The study performed with WebGraph also highlighted, among other facts, the following: similarity was found to be much more concentrated than previously thought; consecutivity is common regarding web graphs. The properties of ordering (and different techniques to produce them) have also been exploited by the same authors to obtain compression with social networks. WebGraph was used in an extensive analysis of many different data sets, which were made available online by the Laboratory for Web Algorithmics [1, 42, 70–72].

**Fig. 3:**
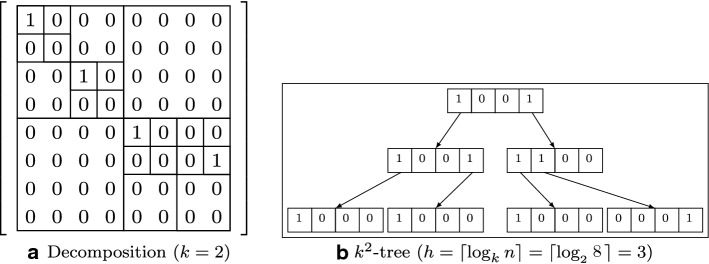
A sample adjacency matrix ($n = |V| = 64$) and corresponding $k^2$-tree representation

The $k^2$-tree is another data structure employed to represent and efficiently store graphs [73]. It may be used to represent static graphs and binary relations in general. It has been used to represent binary relations like web graphs, social networks and RDF data sets by internally using compressed bit vectors. Conceptually, we recursively subdivide each block of a graph’s adjacency matrix until we reach the level of individual cells of the matrix. The idea is to divide (following an MX-Quadtree strategy [74, Sec. 1.4.2.1]) the matrix in blocks and then assign 0 to the block if it only contains zeros (no edges) or 1 if it contains at least an edge. We show in Fig. 3 a sample adjacency matrix on the left and the corresponding $k^2$-tree representation of the decomposition. This representation of the adjacency matrix is actually a $k^2$-tree of height $h = \lceil \log _{k}{n}\rceil$, where ($n = |V|$ and) each node contains a single bit of data. It is 1 for internal nodes and 0 for leaves, except for the last level, in which all nodes are leaves representing values from the adjacency matrix. It is a data structure that also efficiently matches the properties of sparseness and clustering of web graphs.

Another proposal, Log(Graph) [75] is a graph representation that combines high compression ratios with low overhead to enable competitive processing performance while making use of compression. It achieved compression ratios similar to WebGraph while reaching speedups of more than 2x. The authors achieve results by applying logarithm-based approaches to different graph elements. They describe its application on *fine elements* of the adjacency array (the basis of Log(Graph): vertex IDs, offsets and edge weights. From information theory, the authors note that a simple storage lower bound can be the number of possible instances of an entity, meaning the number of bits required to distinguish them. Using this type of awareness on the different elements that represent an adjacency array and by incorporating bit vectors, the authors present a C++ library for the development, analysis and comparison of graph representations composed of the many schemes described in their work.

There were relevant techniques for graph compression the literature on graph compression [76–81] with the WebGraph framework [1, 2] as one of the most well-known, and more recently the $k^2$-tree structure [73, 82–84], only later was the focus cast on being able to represent big graphs with compression while allowing for updates. Furthermore, if we add the possibility of dynamism of the data (the graph is no longer a static object that one wishes to analyse) to the factors guiding representation choice, then it makes sense to think about how to represent a big graph in common hardware not only for storage purposes but also for efficient access with mutability. Works such as VeilGraph [85] approach the concepts of efficient representations by for example incorporating summary graph structures to reduce the total performed computations in the context of graph updates.

A dynamic version of the $k^2$-tree structure was proposed for this purpose [86]. Using compact representations of dynamic bit vectors to implement this data structure, the $k^2$-tree was used to provide a compact representation for dynamic graphs. However, this representation with dynamic compact bit vectors suffers from a known bottleneck in compressed dynamic indexing [87]. It suffers a logarithmic slowdown from adopting dynamic bit vectors. A recent comparative study on the graph operations supported by different $k^2$-tree implementations has also been performed [88]. This work also presented an innovative take on implementing dynamic graphs by employing the $k^2$-tree data structure with a document collection dynamization technique [89], avoiding the bottleneck in compressed dynamic indexing.

## Graph processing: computational units and models

Here we detail the most relevant paradigms and computational units used to express computation in graph processing systems. Programming models for graph processing have been studied and documented in the literature [47, 48]. They define properties such as the granularity of the unit of computation, how to distribute it across the cluster and how communication is performed to synchronize computational state across machines.

### Unit: Vertex-Centric (TLAV)

The vertex-centric paradigm, also known as *think-like-a-vertex* (TLAV), debuted with Google’s Pregel system [8]. An open-source implementation of this model known as Apache Giraph [12] was then offered to the public. Other example systems that were created using that model are GraphLab [90], PowerGraph [60], PowerLyra [61]. As the unit of computation is the vertex itself, the user algorithm logic is expressed from the perspective of vertices. The idea is that a vertex-local function will receive information from the vertex’s incoming neighbours, perform some computation, potentially update the vertex state and then send messages through the outgoing edges of the vertex. A vertex is the unit of parallelization and a vertex program receives a directed graph and a vertex function as input. It was then extended to the concept of vertex scope, which includes the adjacent edges of the vertex. The order of these steps will vary depending on the type of vertex-centric model used (scatter-gather, gather-apply-scatter).

### Model: Superstep Paradigm

In a *superstep* *S*, a user-supplied function is executed for each vertex *v* (this can be done in parallel) that has a status of active. When *S* terminates, all vertices may send messages which can be processed by user-defined functions at step $S+1$.

### Model: Scatter-Gather

Scatter-gather shares the same idea behind vertex-centric but separates message sending from message collecting and update application [91]. In the scatter phase, vertices execute a user-defined function that sends messages along outgoing edges. In the gather phase, each vertex collects received messages and applies a user-defined function to update vertex state.

### Model: Gather-Apply-Scatter

Gather-Sum-Apply-Scatter (GAS) was introduced by PowerGraph [60] and was aimed at solving the limitations encountered by vertex-centric or scatter-gather when operating on power-law graphs. The discrepancy between the ratios of high-degree and low-degree vertices leads to imbalanced computational loads during a superstep, with high-degree vertices being more computationally-heavy and becoming stragglers. GAS consists of decomposing the vertex program in several phases, such that computation is more evenly distributed across the cluster. This is achieved by parallelizing the computation over the edges of the graph. In the gather phase, a user-defined function is applied to each of the adjacent edges of each vertex in parallel.

### Unit: Edge-Centric (TLEV)

The edge-centric approach, also referred as *think-like-an-edge* (TLEV), was popularized by systems like X-Stream [56] and Chaos [62] which specify the computation from the point-of-view of edges. These systems made of use of this paradigm to optimize the usage of secondary storage and network communication with cloud-based machines to process large graphs.

### Unit: Sub-graph-Centric (TLAG)

The previous models are subjected to higher communication overheads due to being fine-grained. It is possible to use sub-graph structure to reduce these overheads (also known as *component-centric* [48]). In this category, the work of [47] denotes two sub-graph-centric approaches: *partition-centric* and *neighbourhood*-*centric*. *Partition-centric* instead of focusing on a collection of unassociated vertices, considers sub-graphs of the original graph. Information from any vertex can be freely propagated within its physical partition, as opposed to the vertex-centric approach where a vertex only accesses the information of its most immediate neighbours. This allows for reduction in communication overheads. Ultimately, the partition becomes the unit of parallel execution, with each sub-graph being exposed to a user function. This sub-graph-centric approach is also known as *think-like-a-graph* [57] (TLAG). *neighbourhood-centric*, on the other hand, allows for a physical partition to contain more than one sub-graph. Shared state updates exchange information between sub-graphs of the same partition, with replicas and messages for sharing between sub-graphs that aren’t in the same partition. For completion, we refer the reader to an analysis of distributed algorithms on sub-graph centric graph platforms [92].

### Model: MEGA

The MEGA model was introduced by Tux2 [93], a system designed for graph computations in machine learning. The model is composed of four functions defined by the user: an *exchange* function which is applied to each edge and can change the value of the edge and adjacent vertices; an *apply* function to synchronize the value of vertices with their replicas; a global *sync* function to perform shared computations and update values shared among partitions; a *mini-batch* function to indicate the execution sequence of other functions in each round.

There are graph processing systems that offer more than one type of model. To achieve parallelism and harness multiple machines in clusters, it is necessary to define how to break down the graph—we provide a high-level overview of methods employed in most well-known graph processing solutions

## Dimension: partitioning

Graph partitioning is an important problem in graph processing, and this importance manifests in two formats. The first, is out of a user’s domain application with the goal of splitting the graph in parts which provide a relevant view of the data. The second, is when partitioning may be considered as a *hyper-algorithm*, that is, it is employed to divide the parts of the graph across a computational infrastructure, typically within the distributed systems’ coordination layer, or across processing units or cores within machines. Machine loads in distributed graph processing systems depend on the way computational units are distributed across workers. The communication between them then depends on the number of units that are replicated, or the number of edges which are cut in the edge-cut partitioning approach. We observe that partitioning has a cyclical nature to itself in the scope of distributed processing: one may wish to execute graph partitioning over a distributed system as part of a domain-specific problem; however, before that graph algorithm can execute, the graph data also incurs partitioning followed by distribution in the underlying (distributed) computational infrastructure. While the study of graph partitioning is not recent, it gained additional depth in the last decade as the number of factors guiding optimization of partitioning increased with the complexity of graph processing systems. We explore partitioning as a relevant dimension to classify graph processing systems as they must approach it in order to enable parallel computation over graphs. The way it is approached becomes a distinctive feature between the systems.

Graph partitioning aims to divide the nodes of the graph into mutually-exclusive groups and to minimize the edges between partitions. This is effectively a grouping of the node set of the graph, which can represent a minimization of communication between partitions, with each partition for example assigned to a specific worker in a distributed system. Partitioning is a task that produces groups of nodes, but grouping nodes is not only achieved with partitioning. We note that other terms exist in the literature such as clustering and community detection. They are not interchangeable, for if a clustering algorithm breaks down the graph into three clusters, it does not necessarily hold true that each cluster represents its own community. As an example, executing a clustering algorithm over a social network graph will result in a number of clusters. If each cluster represents for example a different continent, that does not necessarily mean each cluster represents one single community. Community detection algorithms, on the other hand, consider properties such as the density and interconnections within communities. While clustering and community detection aim to identify similarities between nodes, their underlying assumptions of the graph are not equal, even though proposals have been made to map between these two tasks [94]. Graph clustering shares similarities with graph partitioning in the sense that both produce groups of nodes. However, the objective functions they use are defined differently and subject to different constraints. Graph partitioning, on which we focus, for example, requires that the number of groups (partitions) is known beforehand and is typically subject to more constraints.

**Fig. 4:**
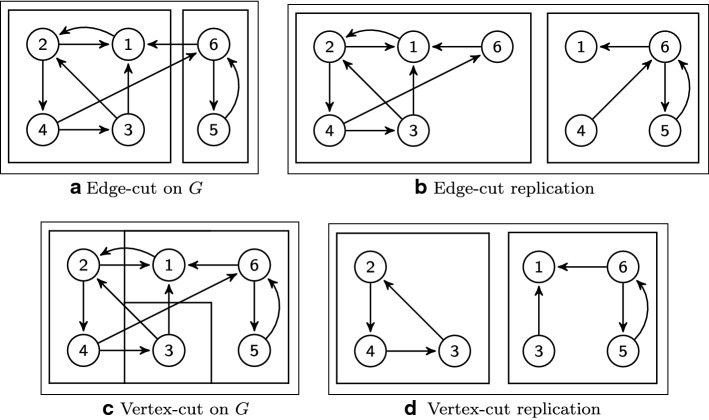
Depiction of vertex-cut and edge-cut over the sample graph *G*

An earlier work on balanced graph partitioning [95] defines the problem as (*k*, *v*)-balanced partitioning: to divide the vertices of the graph into *k* components of almost equal size, with each of size less than $c\ \cdot \frac{n}{k}$ for a given constant $c > 1$. It is a balanced *k*-way partitioning problem which has been studied in the literature [96]. To consider the partitioning problem as a challenge to enabling distributed processing, it is necessary to ask if the goal is to distribute the vertices (edge cut model—*EC*) or the edges (vertex cut model—*VC*) of the graph across machines in order perform it. We provide detail into these problem formulations with an example of vertex-cut and edge-cut in Fig. 4. Furthermore, different combinations between computational unit and cut model are possible: vertex-cut can be used to process in a vertex-centric [97] or edge-centric [60] way, and the same is possible using edge-cut used to partition a graph where computation is vertex-specific [98, 99] or edge-specific.

### Edge-Cut (EC)

Balanced *k*-way partitioning may be defined for edge-cut partitioning, which is associated to vertex-centric (TLAV) systems, the most common computational model in graph processing systems [8, 53, 55]. We reproduce the definition of [50, Sec. 2] for this case, where for a given graph $G = (V, E)$, we wish to find a set of partitions $P = \{P_{1}, P_{2},\dots ,P_{k}\}$. These partitions must be pairwise disjoint and their union is equal to *V* while following these conditions [50]:

2 $$\begin{aligned} \min _{P}&\ | \{ e | e = (v_{i}, v_{j}) \in E, v_{i} \in P_{x}, v_{j} \in P_{y}, x \ne y\} | \end{aligned}$$

3 $$\begin{aligned} s.t.&\ \dfrac{\max _{i}|P_{i}|}{\dfrac{1}{k} \sum _{i=1}^{k} |P_{i}|} \le \epsilon . \end{aligned}$$

Depending on the application objective for which this partitioning type will be performed, Eq. 3 should be adapted. For example, in the case of machines having different characteristics, it should be considered that the load of any machine will be less than the maximum computing power. Or if the graph structure is stored in secondary memory, the interest is on having balanced size partitions with high speed sequential storage access and decreasing the number of cut edges is no longer a focus.

### Vertex-Cut (VC)

In the vertex-cut model, the goal is to distribute edges across partitions. They are placed in different partitions, with vertices being copied in partitions which have their adjacent edges. Care must be taken to balance the number of edges per partition (its measure of size) and to minimize the number of vertex copies. This objective may be formulated as such [50]:

4 $$\begin{aligned} \min _{P}&\ \dfrac{1}{|V|} \sum _{v \in V}{|P(v)|} \end{aligned}$$

5 $$\begin{aligned} s.t.&\ \max _{p_{i}} |\{ e \in E | P(e) = p_{i} \}| \le \epsilon \dfrac{|E|}{k}. \end{aligned}$$

Vertex-cut achieves better performance than edge-cut for natural graphs such as those representing web structure and social networks [50].

### Hybrid-Cut (HC)

Hybrid strategies can be employed to perform the cuts. They can for example be guided with heuristics such as vertex degree in order to decide what to do with them. The PowerLyra [61] system for example allocates the incoming edges of vertices with low degree in a worker. It uses edge-cut for vertices of low-degree and vertex-cut for high-degree vertices.

### Stream-based partitioning

In these methods of partitioning, vertices or edges in the graph are analysed in succession in a stream. Placement decisions are made *online*, that is, when the vertices or edges appear in the stream, and the decisions are based on the location of previous elements. This is done under the assumption that there will be no information on the edges or vertices that will arrive in the flow of the stream. This type of method can rely on edge-cut partitioning (e.g. Random heuristic and the Linear Deterministic Greedy [100], Gemini which uses chunk-based assuming adjacency list model [101], Fennel [102]), vertex-cut partitioning (e.g. Grid heuristic [103], PowerGraph greedy heuristic [60], Graphbuilder [103] placing the edge in the smallest partition, HDRF [104] method which takes into consideration vertex degrees) and there are aspects of these methods that will have different approaches regarding how this is achieved with parallel and distributed execution. Stream-based partitioning is also used as a good choice for loading the graph as it does not have to be fully loaded in memory for partitioning.

### Distributed partitioning

Many distributed partitioning algorithms are based on label propagation [70, 105–107], with variations such as how the specific labelling of a vertex should be influenced by its neighbours, if it should also be influenced by the label’s global representation in the graph and also constraints on the minimum and maximum sizes required for partitions. For example, Revolver, which performs vertex-centric graph partitioning with reinforcement learning, assigns an agent to each vertex, with agents assigning vertices to partitions based on their probability distribution (these are then refined based on feedbacks [97]). The authors of [50] note that other approaches consider the partitioning problem as a multi-objective and multi-constraint problem, achieving better results compared to one-phase methods [108]. Distributed partitioning systems are good for when partitioning is performed once and then calculations are repeatedly performed.

### Dynamic graph partitioning

When the graph is no longer static, vertex and edges may be added or removed as time passes—this is especially true in social networks. This implies that for graphs from which we need to perform computations as they evolve, the original partitioning may become inefficient. With predictable algorithm runtime characteristics, it becomes feasible to keep close the vertices which will be used together in the same supersteps, using for example graph traversal algorithms. But when this is not the case, systems can be designed for example to monitor the load and communication of the machines and migrate vertices as appropriate, with different techniques having been proposed for that purpose (among others, xDGP [109] to repartition massive graphs to adapt to structural changes, GPS [110] which reassigns vertices based on communication patterns, X-Pregel [98] with reduction of message exchanges and dynamic repartitioning). Dynamic partitioning methods have the advantage of outputting very good load balancing and communication cost reductions due to considering heterogeneous hardware and runtime characteristics.

### Partitioning: summary

Employed graph partitioning strategies vary, with different systems offering different solutions. Among performance-impacting factors [50], we have the number of active vertices and edges influencing machine load. At the same time, communication will be more expensive depending on how replication of edges and vertices is performed. Partitioning must balance communication and machine loads. The partitioning challenge in vertex-centric systems is relevant due to how widespread this model is. The authors of [50] note three major approaches for big graph partitioning: *a)* partitioning the graph serially in a single pass and permanently assigning the partition on the first time an edge or vertex is assigned (stream-based); *b)*; methods that partition in a distributed way; *c)* dynamic methods that adapt the partitions based on monitoring the load and communication of machines during algorithm execution. The way the distribution is achieved and data is represented will be a factor in going beyond the *read-eval-write loop*. In this scope, a dynamic method would be necessary as a basis to develop the properties we described. For an in-depth analysis of partitioning methods, vertex cut models and their relation to the dynamic nature of data, we invite the reader to read [50].

Being able to decompose the graph is a cornerstone for efficient and distributed computation of graphs. An equally-important aspect that determines how we must approach the computation is the possible dynamism of the graph. A static graph over which we want to perform analytics is a scenario different from maintaining a large graph available for separate queries and susceptible to updates.

## Dimension: *Dynamism*

We include and consider *dynamism* a relevant dimension of graph processing due to there existing different meanings associated to it in the literature and for which different systems can be attributed. While one may consider static graphs to be completely unrelated to dynamism, there is in fact a relation to it due to what is known as *stream processing*. For example, a graph processing system may ingest an unbounded stream of edges and update statistics over the stream (e.g., keeping a triangle count updated [111]), but stream processing may also take place in static graph processing. This is the case with approaches that process a static graph but process its elements from a stream perspective (e.g., Chaos [62] and X-Stream [56] with their edge-centric approach). Considering if a system targets graphs that change or are immutable (static) is an obvious way to separate graph processing systems when classifying them. However, this dimension is actually a spectrum between the immutable (e.g. stream-based perspectives to process static graphs) and the changing—for example, is the whole graph structure kept in memory (or secondary storage) in a single machine (or across cluster nodes), or is it discarded by proxy of some criteria (and thus one simply updates mathematical properties of the graph using only recent information from the stream)? For this spectrum, the authors of [112] cover definitions found in the literature:

### Temporal graphs

These are, in essence, static graphs which have annotated temporal information which allows for recreating the domain represented by the graph at any given point in time. It is not structurally-changed while doing so; it means that for a given time range or event, only the elements with valid timestamps under required constraints are considered for computation. The work of [113] introduces the temporal graph as a representation encoding temporal data into the graph while retaining the temporal information of the original data. They present metrics that can be used to study temporal graphs and use the representation to explore dynamic temporal properties of data using graph algorithms without requiring data-driven simulations. ImmortalGraph [114] is a storage and execution engine designed with temporal graphs in mind, having achieved greater efficiency than database solutions for graph queries. ImmortalGraph schedules common bulk operations in a way to maximize the benefit of in-memory data locality. It explores the relation between locality, parallelism and incremental computation while enabling mining tasks on temporal graphs. For more information and reach on the topic of temporal graphs, we direct the reader to [115].

### Streaming graph algorithms

[116] With these, the common scenario starts from an empty graph without edges (and a fixed set of vertices). For each algorithm step, a new edge is inserted into the graph or an edge is removed. It is desired that these algorithms are developed to minimize parameters such as graph data structure storage, the time to process an edge or the time to recover the final solution. There exist several systems which process streaming graph computations—we note also for the reader a recent framework for comparing the systems aimed at this type of dynamism [117]. The STINGER data structure has been used for streaming graphs as well [118].

### Sketching and dynamic graph streams

Sketching techniques [119] may be applied to the edge incidence matrix of the input graph to approximate cut structure and connectivity. The idea is to consume a stream of events in order to generate a probabilistic data structure representing properties of the graph.

### Multi-pass streaming graph algorithms

In this type of algorithm, all updates are streamed more than once in order to approximate the computation quality of the solution. Additional complexity can emerge on how the streaming model behaves—it can for example allow for the stream to be manipulated across passes [120] or to stream sorting passes [121].

### Dynamic graph algorithms

For these types, the focus is cast on being able to approximate combinatorial properties of the graph [112] (e.g., connectivity, shortest path distance, cuts, spectral properties) while processing insertions and deletions. The objective with this type of algorithm is to quickly integrate graph updates. Ringo [122] is a single-machine analytics system that supports dynamic graphs.

While partitioning and dynamism are relevant aspects, the scope of graph processing solutions in both industry and academia was shaped by the type of executed workloads.

## Dimension: workload

The type of workload performed by a graph processing system also plays an important role in classifying them. The type of task performed by graph databases is different from the systems that run global algorithms over them. The concept of analysing a graph takes on different contexts depending on user needs. We note that when a graph is to be *processed*, the scope of its data analysis usually falls in these two categories:

(*a*) To retrieve instances of domain-specific relations in the graph (e.g. pattern matching, multi-hop queries). These are usually found in graph databases, with an emphasis on optimization of data query and storage for online transaction processing scenarios. This is often accompanied with the use of graph query languages (GQLs) to execute queries that return a view on the graph and also potentially producing effects on it. (*b*) To execute an algorithm over the whole graph (e.g. PageRank, connected components, detecting communities, finding shortest paths). The solutions for this task, performance-wise, aim to achieve high-performance computational throughput, whether using distributed systems or a single-machine configuration. It is a focus leaning on the data analytics aspect.

The former (*a*) is a common scenario in graph databases such as Neo4j [123] and JanusGraph [124], among others. These databases offer graph query languages (usually even allowing interchangeability between languages) such as Cypher or Gremlin [125]. They are built to store the graph, some with sharding (horizontal scaling) to distribute the graph across the storage/computational infrastructure (some outsource the storage medium to database technologies such as HBase [126] or Cassandra [127]), others in a centralized server (but allowing cluster nodes for the specific purpose of redundancy). They employ schemes to store the graph efficiently while offering transaction mechanisms to operate over the graph and to perform queries. The latter type (*b*) is seen in big (graph) data processing systems like Spark (GraphX library) [13] and Flink (Gelly library) [14]. The mentioned names are all distributed processing frameworks that can take advantage of multi-core machines and clusters. These systems and their libraries allow for expressive computation over graphs in few lines of code. Many of the systems come with their sets of graph algorithms, allowing for the composition of workflows while abstracting away many details from the programmer (regarding distributed computation orchestration and the internal implementation of the graph algorithms).

It is important to consider two definitions regarding the nature of computational tasks: online analytical processing (OLAP) and online transaction processing (OLTP). OLAP is an approach to enable answering multi-dimensional analytical queries quickly. Among its instances we may find tasks such as business reporting for sales, management reporting, business process management [128], financial reporting and others. OLTP, on the other hand, refers to systems that enable and manage transaction-oriented applications, with *transaction* meaning in a computational context the atomic state changes that take place in database systems. OLTP examples include retails sales and financial transaction systems, and applications of this type tend to be high-throughput and update/insertion-intensive in order to provide availability, speed, recoverability and concurrency [129].

The earlier type (*a*) of graph processing task may be associated to OLTP systems as the goal is to store representations of graphs by quickly ingesting new information, efficiently representing it regarding space consumption and access speed, and being able to execute updates under ACID properties (or a subset of those). For this type of task (*a*), one may find numerous graph databases to match the description, such as those for designed for semantic representations, or for property graph models, both and also other specific purposes. The latter type of task (*b*) may be associated to OLAP, where there is a focus on extracting value from the data and the nature of the task is typically read-only. We include graph processing systems (not databases) in this group of OLAP-type tasks, even the systems which support mutability in graphs due to supporting dynamism in any form.

There is a considerable overlap between OLTP-type tasks and graph databases, and there is also an overlap between OLAP-type tasks and graph processing systems. While the distinction between OLAP and OLTP task types is not a dimension that perfectly divides systems in the graph processing landscape, we note that such a distinction holds value in guiding future taxonomies of the graph processing system landscape, and for that reason we include it as a dimension.

The way these three dimensions are accounted for influence the design of graph processing systems. Many different architectures exist, for which we share an exhaustive list of specific solutions, from single-machine systems to parallel processing in clusters and storage in tailor-made graph databases.

## Single-machine and shared-memory parallel approaches

*GraphLab* [130] was published as a framework (implemented in C++) for parallel machine learning and later extended to support distributed settings while retaining strong data consistency guarantees [90]. The authors evaluate it on Amazon EC2, outperforming equivalent MapReduce implementations by over 20X and match the performance of specifically-crafted MPI implementations. GraphLab requires the whole graph and program state to reside in RAM. It uses a chromatic engine so that no adjacent vertices have the same colour and to enable the efficient use of network bandwidth and processor time. The authors evaluate it for applications such as Netflix movie recommendation, video co-segmentation and named entity recognition. It is open-source [131] under the Apache License 2.0.

*GRACE* [132] is a synchronous iterative graph programming model, with separation of application logic and execution policies. Its design includes the implementation (C++) of a parallel execution engine for both synchronous and user-specified asynchronous execution policies. GRACE stores directed graphs, and in its model and the computation is expressed and performed in a way similar to Pregel. It provides additional flexibility, by allowing the user to relax synchronization of computation. This is achieved with user-defined functions which allow updating the scheduling priority of vertices that receive messages (the vertex order in which computation will take place within an iteration). GRACE’s design targets both shared-memory and distributed system scenarios, but the initial prototype focuses on shared-memory. We did not find the source code available.

*Ligra* [133] is a C++ lightweight graph processing framework targeting shared-memory parallel/multi-core machines, easing the writing of graph traversal algorithms. This framework offers two map primitives to operate a given logic on vertices (VertexMap) and edges (EdgeMap) and supports two data types: the traditional graph $G = (V, E)$ as we described in an earlier section, and another one to represent subsets of vertices. The interface is designed to enable the processing of edges in different orders depending on the situation (as opposed to Pregel or Giraph). The code of Ligra represents in-edges and out-edges as arrays, with in-edges for all vertices being partitioned by their target vertex and storing the source vertices, and the out-edges are in an array partitioned by source vertices and storing the target vertices. While the authors claim to have achieved good performance results, they mention Ligra does not support algorithms based on modifying the input graph. It is available [134] under the MIT License.

*Ringo* [122] is an approach for multi-core single-machine big-memory configurations. It is a high-performance interactive analytics system using a Python front-end on a scalable parallel C++ back-end, representing the graph as a hash table of nodes. It supports fast execution times with exploratory and interactive use, offering graph algorithms in a high-level language and rich support for transformations of input data into graphs. Ringo is open-source and available [135] under the BSD License.

*Polymer* [136] is a NUMA-aware graph analytics system on multi-core machines that is open-source [137] under the Apache License 2.0 and implemented in C++. It innovated by differentially allocating and placing topology data, application-defined data and mutable run-time graph system states according to access patterns to minimize remote accesses. Polymer also deals with random accesses by converting the random ones into sequential remote accesses using lightweight vertex replication across the NUMA nodes. It was built with a hierarchical barrier for increased parallelism and locality. The design also includes edge-oriented balanced partitioning for skewed graphs and adaptive data structures in function of the fraction of active vertices. It was compared to Ligra, X-Stream and Galois on an 80-core Intel machine (no hyper-threading) and on a 64-core AMD machine. For different algorithms across several data sets, Polymer consistently almost always achieved the lowest execution time.

*GraphMat* [138] is a framework written in C++ aimed at bridging the user-friendly graph analytics and native hand-optimized code. It presents itself as a vertex-centric framework without sacrificing performance, as it takes vertex programs and maps them to exclusively use sparse matrix high-performance back-end operations. GraphMat takes graph algorithms expressed as vertex programs and performs generalized sparse matrix vector multiplication on them. It achieved greater performance than other frameworks such as 5-7X faster than GraphLab, Galois and ComBLAS. It also achieved multi-core scalability, being over 10X faster than single-threaded implementation on a 24-core machine. It is open-source and available [139] under specific conditions by Intel.

*Mosaic* [140] is a system for single heterogeneous machines with fast storage media (e.g., NVMe and SSDs) and massively-parallel co-processors (e.g., Xeon Phi) developed to enable the processing of trillion-edge graphs. The system is designed explicitly separating graph processing engine components into scale-up and scale-out goals. It is written in C++ uses a compact representation of the graph using Hilbert-ordered tiles for locality, load balancing and compression and uses a hybrid computation model that uses both vertex-centric operations (on host processors) and edge-centric operations (on co-processors). Mosaic is open-source [141] under the MIT License.

## High-performance computing

These systems are hallmarks of high-performance computing solutions applied to graph processing. Their merits encompass algebraic decomposition of the major graph operations, implementing them and translating them across different homogeneous layers of parallelism (across cores, across CPUs). Here we mention what are, to the best of our knowledge, the most relevant works:

*Parallel Boost Graph Library* (PBGL) [142] . It is an extension (C++) of Boost’s graph library. It is a distributed graph computation library, also offering abstractions over the communication medium (e.g. MPI). The graph is represented as an adjacency list that is distributed across multiple processors. In PBGL, vertices are divided among the processors, and each vertex’s outgoing edges are stored on the processor storing that vertex. PBGL was evaluated on a system composed of 128 compute nodes connected via Infiniband. It is available [143] under a custom Boost Software License 1.0.

*CombBLAS* [144]. A parallel graph distributed-memory library in C++ offering linear algebra primitives based on sparse arrays for graph analytics. This system considers the adjacency matrix of the graph as a sparse matrix data structure. CombBLAS is edge-based in the sense that each element of the matrix represents an edge and the computation is defined over it. It decouples the parallel logic from the sequential parts of the computation and makes use of MPI. However, its MPI implementation does not take advantage of flexible shared-memory operations. Its authors targeted hierarchical parallelism of supercomputers for future work. It is available [145] under a custom license.

*HavoqGT* [146] is a C++ system with techniques for processing scale-free graphs using distributed memory. To handle the scale-free properties of the graph, it uses edge list partitioning to deal with high-degree vertices (hubs) and dummy vertices to represent them to reduce communication hot spots. HavoqGT allows algorithm designers to define vertex-centric procedures in a distributed asynchronous visitor queue. This queue is part of an asynchronous visitor pattern designed to tackle load imbalance and memory latencies. HavoqGT targets supercomputers and clusters with local NVRAM. It is available [147] under the GNU Lesser General Public License 2.1.

## Distributed graph processing systems

While the previous systems we detailed performed analytics and enabled the execution of graph algorithms, they did so with a focus on specific hardware and distributed memory. We list here some of the most relevant state-of-the-art systems used for graph processing in the scope of analytics (OLAP). Their use of different architectures (from using local commodity clusters to cloud-based execution) and greater flexibility of deployment scenarios differentiate them from those of the previous section. The following systems are relevant names in the literature, with Giraph being the first open-source implementation of the Pregel approach to graph processing, and Spark and Flink being open-source general distributed processing systems with graph processing APIs:

*Apache Giraph* [12] is an open-source Java implementation of Pregel [8], tailor-made for graph algorithms, supporting the GAS model and licensed [148] under the Apache License 2.0. It was created as an efficient and scalable fault-tolerant implementation on clusters with thousands of commodity hardware, hiding implementation details underneath abstractions. Work has been done to extend Giraph from the *think-like-a-vertex* (TLAV) model to *think-like-a-graph* (TLAG) [57]. It uses Hadoop’s MapReduce implementation to process graphs. Giraph [148] allows for master computation, sharded aggregators (relevant when computing a final result comprised of intermediate data from nodes), has edge-oriented input, and also uses out-of-core computation—limited partitions in memory. Partitions are stored in local disks, and for cluster computing settings, the out-of-core partitions are spread out across all disks. Giraph attempts to keep vertices and edges in memory and uses only the network for the transfer of messages. Improving Giraph’s performance by optimizing its messaging overhead has also been studied [149]. It is interesting to note that single-machine large-memory systems such as Ringo highlight the message overhead as one of the major reasons to avoid a distributed processing scheme.

*Naiad* is an open-source [150] (Apache License 2.0) dataflow processing system [151] offering different levels of complexity and abstractions to programmers. It allows programmers to implement graph algorithms such as weakly connected components, approximate shortest paths and others while achieving better performance than other systems. Naiad is implemented in C# and allows programmers to use common high-level APIs to express algorithm logic and also offers a low-level API for performance. Its concepts are important and other systems could benefit from offering tiered programming abstraction levels as in Naiad. Its low-level primitives allow for the combination of dataflow primitives (similar to those VeilGraph uses from Flink) with finer-grained control on iterative computations. An extension to Flink’s architecture to offer this detailed control would enrich the abilities that our framework is able to offer to users.

*Apache Flink* [14], formerly known as Stratosphere [152], it is a framework which supports built-in iterations [14] (and delta iterations) to efficiently aid in graph processing and machine learning algorithms. It is licensed [153] under the Apache License 2.0 and has a graph processing API called Gelly, which comes packaged with algorithms such as PageRank, single-source shortest paths and community detection, among others. Flink supports Java, Python and Scala. It explicitly supports three vertex-based programming models: *think-like-a-vertex* (TLAV) described as the most generic model, supporting arbitrary computation and messaging for each vertex; Scatter-Gather, which separates the logic of message production from the logic of updating vertex values, which may typically make these programs have lower memory requirements (concurrent access to the inbox and outbox of a vertex is not required) while at the same time potentially leading to non-intuitive computation patterns; Gather-Sum-Apply-Scatter (GAS), which is similar to Scatter-Gather but the Gather phase parallelizes the computation over the edges, the messaging phase distributes the computation over the vertices and vertices work exclusively on neighbourhood, where in the previous two models a vertex can send a message to any vertex provided it knows its identification. It supports all Hadoop file systems as well as Amazon S3 and Google Cloud storage, among others. Delta iterations are also possible with Flink, which is quite relevant as they take advantage of computational dependencies to improve performance. It also has flexible windowing mechanisms to operate on incoming data (the windowing mechanism can also be based on user-specific logic). Researchers have also looked into extending its DataStream constructs and its streaming engine to deal with applications where the incoming flow of data is graph-based [154].

*Apache Spark* [155] and its GraphX [13] graph processing library, licensed [156] under the Apache License 2.0. It is a graph processing framework built on top of Spark (a framework supporting Java, Python and Scala), enabling low-cost fault-tolerance. The authors target graph processing by expressing graph-specific optimizations as distributed join optimizations and graph views’ maintenance. In GraphX, the property graph is reduced to a pair of collections. This way, the authors are able to compose graphs with other collections in a distributed dataflow framework. Operations such as adding additional vertex properties are then naturally expressed as joins against the collection of vertex properties. Graph computations and comparisons are thus an exercise in analysing and joining collections.

*GraphTau* [157] is a time-evolving graph processing framework on top of Spark (Java, Scala). It represents computations on time evolving graphs as a stream of consistent and resilient graph snapshots and a small set of operators that manipulate such streams. GraphTau builds fault-tolerant graph snapshots as each small batch of new data arrives. It is also able to periodically load data from graph databases and reuses many operators from GraphX and Spark Streaming. For algorithms (based on label propagation) that are not resilient to graph changes, GraphTau introduced an online rectification model, where errors caused by underlying graph modifications are corrected in online fashion with minimal state. Its API frees programmers from having to implement graph snapshot generation, windowing operators and differential computation mechanisms. We did not find its source code available.

*Tink* [158] is a library for distributed temporal graph analytics. It is built on Flink (Java, Scala) and focuses on *interval* graphs, where each edge has an associated starting time and ending time. The author created different graphs with information provided by Facebook and Wikipedia in order to evaluate the framework. Tink defines a temporal property graph model. It is available online [159], although we did not find information pertaining licensing.

To the best of our knowledge, currently both Flink and Spark are the most widely-known distributed processing frameworks (we note GraphTau, although its code is not available, is built over Spark) based on dataflow programming. While the use of dataflows grants flexibility to program implementation and execution by decoupling the program logic from how it is translated to the workers of a cluster, the graph libraries of these systems do not allow in an efficient way for a graph to be updated using stream-processing semantics while also maintaining the graph structure during computation. It is possible to update graphs using these systems, but they make use of batch processing APIs for which the dataflow graphs must not become excessively big (or else dataflow plan optimizers may be *locked* in the phase of exploring the optimization space of the execution plan) and graph must be periodically written to secondary storage (as a solution to avoid having progressively bigger execution plans).

Flink’s Gelly library has been used in GRADOOP, which is an open-source [160] (Apache License 2.0) distributed graph analytics research framework [161] under active development and providing higher-level operations. GRADOOP extends Gelly with additional specialized operators such as a graph pattern matching operator (which abstracts a cost-based query engine) and a graph grouping operator (implemented as a composition of map, filter, group and join transformations on Flink’s DataSet). GRADOOP also adopts the Cypher query language (typically found in graph databases like Neo4j) to express logic that is translated to the relational algebra that underlies Flink’s DataSet [162].

In a similar way, Spark has its graph processing library GraphX which was built over the system’s batch processing API, like the case of Flink’s Gelly and also suffering from the same previously mentioned limitations. A higher-level API was designed to extend the functionalities of GraphX while harnessing Spark’s DataFrame API. For this, the GraphFrames open-source [163] (Apache License 2.0) library was created [164]. A look at its implementation reveals that it has less high-level operations than Gelly. Effectively, without simulating some of Gelly’s API, equivalent programs in GraphX lend themselves to more conceptual verbosity due to the lack of syntactic sugar.

**Fig. 5:**
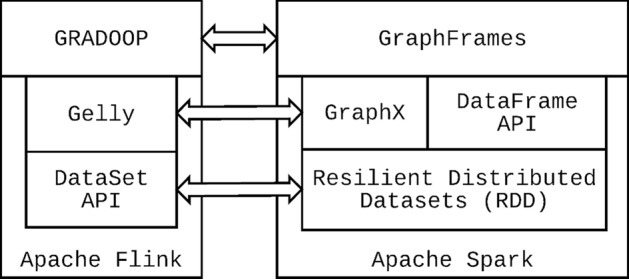
Contrast of the Flink and Spark distributed dataflow ecosystems for graph processing

We display in Fig. 5 parallels between Flink, Spark and the graph processing ecosystems built on top of them. Gelly’s equivalent in Spark is GraphX, implemented in Scala. Vertices and edges are manipulated by using Spark’s Resilient Distributed Datasets (RDDs), which can be viewed as a conceptual precursor to Flink’s DataSet. Spark also offers the DataFrame API to enable tabular manipulation of data. GraphFrames is another graph processing library for Spark. While it has interoperability and a certain overlap with the functionality offered in GraphX, it integrates the tabular perspective supported by Spark’s DataFrame API and also supports performing traversal-like queries of the graph via SparkSQL. In this way, GraphFrames provides graph analytics capabilities in Spark much the same way GRADOOP does in Flink.

The next two examples, X-Stream and Chaos are grouped together as they brought relevance to the edge-centric (TLAE) model and employed it to explore novel ways to balance network latencies and use of SSDs to increase performance:

*X-Stream* [56]. A system that provided an alternative view to the traditional *vertex-centric* approach. It is based on considering computation from the perspective of edges instead of vertices and experiments optimized the use of storage I/O both locally and on the cloud. X-Stream is an open-source system written in C++ which introduced the concept of *edge-centric* graph processing via streaming partitions. X-Stream exposes an edge-centric scatter-gather programming model that was motivated by the lack of access locality when traversing edges, which makes it difficult to obtain good performance. State is maintained in vertices. This tool uses the streaming partition, which works well with RAM and secondary (SSD and Magnetic Disk) storage types. It does not provide any way by which to iterate over the edges or updates of a vertex. A sequential access to vertices leads to random access of edges which decreases performance. X-Stream is innovative in the sense that it enforces sequential processing of edges (edge-centric) in order to improve performance. It is available [165] under the Apache License 2.0.

*Chaos* [62]. A system written in C++ which had its foundations on XStream. On top of the secondary storage studies performed in the past, graph processing in Chaos achieves scalability with multiple machines in a cluster computing system. It is based on different functionalities: load balancing, randomized work stealing, sequential access to storage and an adaptation of X-Stream’s streaming partitions to enable parallel execution. Chaos is composed of a storage sub-system and a computation sub-system. The former exists concretely as a storage engine in each machine. Its concern is that of providing edges, vertices and updates to the computation sub-system. Previous work on X-Stream highlighted that the primary resource bottleneck is the storage device bandwidth. In Chaos, the storage and computation engines’ communication is designed in a way that storage devices are busy all the time—thus optimizing for the bandwidth bottleneck. It was released [166] under the Apache License 2.0.

The following graph processing systems were grouped together because each of the improvements they proposed are important concerns to be aware of in designing graph processing systems.

*PowerLyra* [61] is a graph computation engine written in C++ which adopts different partitioning and computing strategies depending on vertex types. The authors note that most systems use a *one-size-fits-all* approach. They note that Pregel and GraphLab focus in hiding latency by evenly distributing vertices to machines, making resources locally accessible. This may result in imbalanced computation and communication for vertices with higher degrees (frequent in scale-free graphs). Another provided design example is that of PowerGraph and GraphX which focus on evenly parallelizing the computation by partitioning edges among machines, incurring communication costs on vertices, even those with low degrees. PowerLyra was released under the Apache License 2.0 [167].

*Kineograph* [168] is a system which combines snapshots allowing full processing in the background and explicit alternative/custom functions that, besides assessing updates’ impact, also apply them incrementally, propagating their outcome across the graph. It is a distributed system to capture the relations in incoming data feeds, built to maintain timely updates against a continuous flow of new data. Its architecture uses *ingest* nodes to register graph update operations as identifiable transactions, which are then distributed to *graph* nodes. Nodes of the latter type form a distributed in-memory key/value store. Kineograph performs computation on static snapshots, simplifying algorithm design. We did not find its source code online.

*Tornado* [169] is a system for real-time iterative analysis over evolving data. It was implemented over Apache Storm and provides an asynchronous bounded iteration model, offering fine-grained updates while ensuring correctness. It is based on the observations that: *1)* loops starting from *good enough* guesses usually converge quickly; *2)* for many iterative methods, the running time is closely related to the approximation error. From this, an execution model was built where a main loop continuously gathers incoming data and instantly approximates the results. Whenever a result request is received, the model creates a branch loop from the main loop. This branch loop uses the most recent approximations as a guess for the algorithm. We did not find its source code online.

*KickStarter* [170] is a system that debuted a runtime technique for trimming approximation values for subsets of vertices impacted by edge deletions. The removal of edges may invalidate the convergence of approximate values pertaining monotonic algorithms. KickStarter deals with this by identifying values impacted by edge deletions and adapting the network impacts before the following computation, achieving good results on real-world use-cases. Despite this, by focusing on monotonic graph algorithms, its scope is narrowed to selection-based algorithms. For this class, updating a vertex value implies choosing a neighbour under some criteria. KickStarter is now known as GraphBolt, a recent work [171, 172] licensed under the MIT License [171] which offers a generalized incremental programming model enabling development of incremental versions of complex aggregations. Both were written in C++.

*Pixie* [173] is a graph-based scalable real-time recommendation system used at Pinterest. Using a set of user-specific *pins* (in Pinterest, users have boards in which they store pins, where each pin is a combination of image and text), Pixie chooses in real-time the pins that are most related to the query, out of billions of candidates. With this system, Pinterest was able to execute a custom (Pixie Random Walk) algorithm on an object graph of 3 billion vertices and 17 billion edges. On a single server, they were able to serve around 1200 recommendation requests per seconds with 60 millisecond latency. The authors note that the deployment of Pixie benefited from large RAM machines, using a cluster of Amazon AWS r3.8xlarge machines with 244GB RAM. They fitted the pruned Pinterest graph (3 billion vertices, 17 billion edges) in around 120GB of RAM, in a setup that yielded the following advantages: random walk not forced to cross machines, which increases performance; multiple walks can be executed on the graph in parallel; the system can be parallelized and scaled by adding more machines to the cluster. This system is a relevant case study (of applying graph theory to recommendation systems at scale) as a scalable system for processing on large graphs a biased random walk algorithm (with user-specific preferences) while using graph pruning techniques to disregard large boards that are too diverse and diffuse the random walk (the non-pruned graph has 7 billion vertices and 100 billion edges). We did not find the source code available online.

*FlowGraph* [174] is a system that proposes a syntax for a language to detect temporal patterns in large-scale graphs and introduces a novel data structure to efficiently store results of graph computations. This system is a unification of graph data with stream processing considering the changes of the graph as a stream to be processed and offering an API to satisfy temporal patterns. We did not find its source code available.

*GPS* [110] is an open-source (BSD License) scalable graph processing system written in Java and allowing fault-tolerant and easy-to-program algorithm execution on very large graphs. It adopts Pregel’s vertex-centric API and extends it with: features to make global computations easier to express and more efficient; dynamic repartitioning scheme to reassign vertices to different workers during computation based on messaging patterns; distribution of high-degree vertex adjacency lists across all computer nodes to improve performance (something that PowerGraph and PowerLyra later adopted). It was designed to run on a cluster of machines such as Amazon EC2, over which the authors tested their work. GPS’s initial version was run on up to 100 Amazon EC2 large instances and on graphs of up to 250 million vertices and 10 billion edges. It is open-source and available under the BSD License [175].

*GoFFish* [176] is a sub-graph centric programming abstraction and framework co-designed with a distributed persistent graph storage for large scale graph analytics on commodity clusters, aiming to combine the scalability of the vertex-centric (TLAV) approach with flexibility of shared-memory sub-graph computation (TLAG). It is written in Java. GoFFish states that two sub-graphs many not share the same vertex, but they can have remote edges that connect their vertices, provided that the sub-graphs are on different partitions. If two sub-graphs in the same partition share an edge, by definition they are merged into a single-sub-graph. It was evaluated with a cluster of 12 nodes each with 8-core Intel Xeon CPUs, 16 GB RAM and 1 TB SATA HDD. The authors compare the execution of GoFFish (one worker per node) with Giraph (default two workers per node), achieving faster execution times for algorithms such as PageRank, connected components and single-source shortest-paths. Its source code is available though we did not find any information pertaining licensing. While its source code is available [177], we did not find information regarding licensing.

*FBSGraph* [178] presents a forward and backward sweeping execution method to accelerate state propagation for asynchronous graph processing. In asynchronous graph processing, each vertex maintains a state which can be asynchronously updated in an iterative fashion. The method presented in FBSGraph relies on the observation that state can be propagated faster by processing vertices sequentially along the graph path in each round. They achieve greater execution speed when analysing several graph algorithms across a set of datasets, comparing against systems such as PowerGraph and GraphLab. We did not find its source available.

*GrapH* [179] is a graph processing system written in Java that uses vertex-cut graph partitioning that takes into consideration the diversity of vertex traffic and the heterogeneous costs of the network. It relies on a strategy of adaptive edge migration to reduce the frequency of communication across expensive network links. For this work, the authors focused on vertex-cut as it has better partitioning properties for real-world graphs that have power-law degree distributions. GrapH has two partitioning techniques, *H-load* which is used for the initial partitioning of the graph so that each cluster worker can load it into local memory, and *H-adapt*, a distributed edge migration algorithm to address the dynamic heterogeneity-aware partitioning problem. In evaluation, GrapH outperformed PowerGraph’s vertex-cut partitioning algorithm with respect to communication costs. While its source code is available [180], we found no information on licensing.

*Julienne* [181] is built over Ligra (C++) and provides an interface to maintain a collection of buckets under vertex insertions and bucket deletions. They evaluated under bucketing algorithms such as weighted breadth-first search, *k*-core and approximate set cover. The authors describe as *bucketing-based* algorithms those that maintain vertices in a set of unordered buckets—and in each round, the algorithm extracts the vertices contained in the lowest (or highest) bucket to perform computation on them. Then, it can update the buckets containing the extracted vertices or their neighbours. For example, weighted breadth-first search processes vertices level by level, where level *i* contains all vertices at distance *i* from the source vertex. The system was tested in a multi-core machine with 72 cores (4 CPUs at 2.4GHz) and 1TB of main memory, achieving performance improvements on several data sets when compared to systems such as Galois, base Ligra and Galois. We did not find its source code available.

*GraphD* [182] is an out-of-core system inspired by Pregel and targeting efficient big graph processing using a small cluster of commodity machines connected by Gigabit Ethernet, contrasting with other out-of-core works that focus on specialized hardware. The authors focus on a setting that sees vertex-centric programs being data-intensive, as the CPU cost of computing a message is small when compared to the network transmission cost. GraphD masks disk I/O overhead with message transmission though parallelism of computation and communication. It eliminates the need for (expensive) external-memory join or group-by operations, which are required in other systems such as Chaos. It was evaluated on PageRank, single-source shortest-paths and connected components. GraphD was evaluated against distributed out-of-core systems Pregelix, HaLoop and Chaos, against single-machine systems GraphChi and X-Stream and representative in-memory systems Pregel and Giraph, achieving better performance in some scenarios. We did not find its source available.

*TurboGraph++* [183] is a graph analytics system that exploits external memory for scale-up without compromising efficiency. It introduced an abstraction called nested windowed streaming to achieve scalability and increase efficiency in processing neighbourhood-centric analytics (in which the total size of neighbourhoods around vertices can exceed the available memory budget). This streaming model regards a sequence of vertex values and an adjacency list stream. The goal is to efficiently support the *k*-walk neighbourhood query (a class of graph queries defined by the authors, where walks are enumerated and then computation is done for each one) with fixed size memory. In the model, during user computation, they define an update stream as the sequence of updates generated to the ending vertex of each walk, with each update represented as a pair of target vertex ID and update value. TurboGraph++ has the goal of balancing the workloads across machines, which requires balancing the number of edges and the number of high-degree and low-degree vertices among machines. It also focuses on balancing the number of vertices on each machine so that each one requires the same memory budget. We did not find its source code available online.

*GraphIn* [184] is a dynamic graph analytics framework proposed to handle the scale and evolution of real-world graphs. It aimed to improve over approaches to processing dynamic graphs which repeatedly run static graph analytics on stored snapshots. GraphIn proposes an adaptation of gather-apply-scatter (GAS) called I-GAS which enables the implementation of incremental graph processing algorithms across multiple CPU cores. It also introduces an optimization heuristic to choose between static or dynamic execution based on built-in and user-defined graph properties. Native and benchmarking code were implemented in C++ and for experimental evaluation it was compared to GraphMat and STINGER. The heuristic-base computation made GraphIn faster than systems using fixed strategies. We did not find its source code available.

The following works focus on specific techniques such as using specific hardware such as SSDs or GPUs. We first list frameworks and systems that were proposed in the last years to use the single-instruction multiple-data (SIMD) capabilities of GPUs for graph processing:

*MapGraph* [185] is a high-performance parallel graph programming framework, able to achieve up to 3 billion traversed edges per second using a GPU. It represents the graph with a compressed sparse row (CSR) data structure and chooses different scheduling strategies depending on the size of the *frontier* (the set of vertices that are active in a given iteration). It encapsulates the complexity of the GPU architecture while enabling dynamic runtime decisions among several optimization strategies. Users need only to write sequential C++ code to use the framework. The early MapGraph work was first available as an open-source project [186] licensed under the Apache License 2.0, but it has been integrated in the former line of products of Blazegraph, also available online [187].

*CuSha* [188] is a CUDA-based graph processing framework written in C++ which was motivated by the negative impact that irregular memory accesses have on the compressed sparse row graph (CSR) representation. CuSha overcomes this by: *1)* organizing the graph into autonomous sets of ordered edges called *shards* (a representation they call *G-Shards*) unto which GPU hardware resources are mapped for fully coalesced memory accesses; *2)* accounting for input graph properties such as sparsity (the sparser the graph, the smaller the computation windows) to avoid GPU under-utilization (*Concatenated Windows*, or *CW*). This framework allows users to define vertex-centric algorithms to process large graphs on GPU. It is open-source [189] and available under the MIT License.

*Gunrock* [190, 191] is an open-source [192] (Apache License 2.0) CUDA library for graph processing targeting the GPU and written in C. It implements a data-centric abstraction focused on operations on a vertex or edge frontier. For different graph algorithms, it achieved at least an order of magnitude speedup over PowerGraph and better performance than any other high-level GPU graph library at the time. Its operations are bulk-synchronous and manipulate a frontier, which is a subset of the edges or vertices within the graph that is relevant at a given moment in the computation. Gunrock couples high-performance GPU computing primitives and optimization strategies with a high-level programming model to quickly develop new graph primitives. It was evaluated using breadth-first search, depth-first search, single-source shortest paths, connected components and PageRank.

*Lux* [193] is a distributed multi-GPU system written in C++ for fast graph processing by exploiting aggregate memory bandwidth of multiple GPUs and the locality of the memory hierarchy of multi-GPU clusters. It proposes a dynamic graph repartitioning strategy to enable well-balanced distribution of workload with minimal overhead (improving performance by up to 50%), as well as a performance model providing insight on how to choose the optimal number of nodes and GPUs to optimize performance. Lux is aimed at graph programs that can be written with iterative computations. Vertex properties are read-only in each iteration, with updates becoming visible at the end of an iteration. It offers two execution models: *pull* which optimizes run-time performance of GPUs (enables optimizations like caching and locally aggregating updates in GPU shared memory); and *push*, which optimizes algorithmic efficiency (maintains a frontier queue and only performs computation over the out-edges of vertices in the frontier). Its source code is available [194] under Apache License 2.0.

*Frog* [195] is a light-weight asynchronous processing framework written in C. The authors note that common colouring algorithms may suffer from low parallelism due to a large number of colours being needed to process large graphs with billions of vertices. Frog separates vertex processing based on colour distribution. They propose an efficient hybrid graph colouring algorithm, relying on a relaxed pre-partition method to solve vertex classification with a lower number of colours, without forcing all adjacent vertices to be assigned different colours. The execution engine of Frog scans the graph by colour, and all vertices under the same colour are updated in parallel in the GPU. For large graphs, when processing each partition, the data transfers are overlapped with GPU kernel function executions, minimizing PCIe data transfer overhead. It is open-source [196] and licensed under the GNU General Public License 2.0.

*Aspen* [197] is a graph-streaming extension of the Ligra interface, supporting graph updates. To support this, the authors developed and presented the *C*-tree data structure which achieves good cache locality, lowers space use and has operations which are efficient from a theoretical perspective. It applies a chunking scheme over the tree, storing multiple elements in a tree-node. The scheme takes the ordered set of elements that are represented. More relevant elements are stored in tree nodes, while the remaining ones are associated in tails of the tree nodes. It employs compression and supports parallelism. The authors evaluate it with the largest publicly-available graph, which has more than two hundred billion edges on a multi-core server with 1 TB memory. Source code is available online [198] albeit no license information was provided.

*Gluon* [199] was introduced as a new approach to create distributed-memory graph analytics systems able to use heterogeneity in partitioning policies, processor types (GPU and CPU) and programming models. To use Gluon, programmers implement applications in shared-memory programming systems of their choice and then interface the applications with Gluon to enable execution on heterogeneous clusters. Gluon optimizes communication by taking advantage of temporal and structural invariants of graph partitioning policies. It runs on shared-memory NUMA platforms and NVIDIA GPUs. Its programming model offers a small number of programming patterns implemented in C++, its library offers concurrent data structures, schedulers and memory allocators and the runtime executes programs in parallel, using parallelization strategies as optimistic and round-based execution. Gluon is available [200] under the 3-Clause BSD License.

*Hornet* [201] is a data structure for efficient computation of dynamic sparse graphs and matrices using GPUs. It is platform-independent and implements its own memory allocation operation instead of standard function calls. The implementation uses an internal data manager which makes use of block arrays to store adjacency lists, a bit tree for finding and reclaiming empty memory blocks and $B^{+}$ trees to manage them. It was evaluated using an NVIDIA Tesla GPU and experiments targeted the update rates it supports, algorithms such as breadth-first search (BFS) and sparse matrix-vector multiplication. Hornet is available [202] under the 3-Clause BSD License.

*faimGraph* [203] introduced a fully-dynamic graph data structure performing autonomous memory management on the GPU. It enables complete reuse of memory and reduces memory requirements and fragmentation. The implementation has a vertex-centric update scheme that allows for edge updating in a lock-free way. It reuses free vertex indices to achieve efficient vertex insertion and deletion, and does not require restarting as a result of a large number of edge updates. faimGraph was benchmarked against Hornet on an NVIDIA GeForce GTX Titan Xp GPU using algorithms such as PageRank and triangle counting. Source code is available online [204] without a specified license.

*GraphCage* [205] is a cache-centric optimization framework to enable highly efficient graph processing on GPUs. It was motivated by the random memory accesses which are generated by sparse graph data structures, which increase memory access latency. The authors note that conventional cache-blocking suffers from repeated accesses when processing large graphs on GPUs, and propose a throughput-oriented cache blocking scheme (*TOCAB*). GraphCage applies the scheme to both push and pull directions and coordinates with load balancing strategies by considering sparsity of sub-graphs. This technique is applied to traversal-based algorithms by considering the benefit and overhead in different iterations with working sets of different sizes. In its evaluation, GraphCage achieved in average lower execution times for one PageRank iteration compared to both Gunrock and CuSha. We did not find its source code available.

For more information on GPU use cases for graph processing approaches, we point the readers to [206].

*FlashGraph* [207] is a graph processing engine implemented in C++ over a user-space SSD file system designed for high IOS and very high levels of parallelism. Vertex state is stored in memory while edge lists are on SSDs. Latency is hidden by overlapping computation with I/O, a concept similar to X-Stream and Chaos, and edges lists are only accessed if requested by applications from SSDs. FlashGraph has a vertex-centric (TLAV) interface, its designed to reduce CPU overhead and increase throughput by conservatively merging I/O requests, and the authors demonstrate that FlashGraph in semi-external memory executes many algorithms with a performance of up to 80% of the in-memory implementation and It also outperformed PowerGraph. It is open-source [208] under the Apache License 2.0.

*GraphSSD* [209] is a semantic-aware SSD framework and full system solution to store, access and execute graph analytics. Instead of considering storage as a set of blocks, it accounts for graph structure while choosing graph layout, access and update mechanisms. GraphSSD innovates by considering a vertex-to-page mapping scheme and uses advanced knowledge of flash properties to reduce page accesses. It offers a simple API to ease development of applications accessing graphs as native data and its evaluation showcased average performance gains for basic graph data fetch functions on breadth-first search, connected components, random-walk, maximal independent set and PageRank. We did not find its source available.

In Table 1 we summarize distinguishing features and licenses for the graph processing systems detailed in this section. The last reference in front of every system name is its open-source code repository, when available. The second group from the top (PBGL, CombBLAS and HavoqGT) contains systems which use multiple machines for computation but not in the typical cluster scenario. Instead, they are characterized by using specific machines for high-performance computing.

**Table 1:** Summary of graph system distinctive features

System	Multi-core	GPU	Cluster	Languages	License	Notes
GraphLab [130, 131]	$\cdot$		$\cdot$	C++	AL 2.0	*N/A*
GRACE [132]	$\cdot$		$\cdot$	C++	*Unavailable*	*N/A*
Ligra [133, 134]	$\cdot$			C++	MIT	*N/A*
Ringo [122, 135]	$\cdot$			C++, Python	BSD	*N/A*
Polymer [136, 137]	$\cdot$			C++	AL 2.0	*N/A*
GraphMat [138, 139]	$\cdot$			C++	*Custom*	*N/A*
Mosaic [140, 141]	$\cdot$			C++	MIT	*Fast storage*
PBGL [142, 143]	$\cdot$			C++	*Custom*	*Hardware*
CombBLAS [144, 145]	$\cdot$			C++	*Custom*	*Hardware*
HavoqGT [146, 147]	$\cdot$			C++	GNU LGPL 2.1	*Hardware*
Apache Giraph [12, 148]	$\cdot$		$\cdot$	Java	AL 2.0	*N/A*
Naiad [150, 151]	$\cdot$			C#	AL 2.0	*N/A*
Apache Flink [14, 153]	$\cdot$		$\cdot$	Java, Python, Scala	AL 2.0	*N/A*
Apache Spark [155, 156]	$\cdot$		$\cdot$	Java, Python, Scala	AL 2.0	*N/A*
GraphTau [157]	$\cdot$		$\cdot$	Java, Scala	*Unavailable*	*N/A*
Tink [158, 159]	$\cdot$		$\cdot$	Java, Scala	AL 2.0	*N/A*
X-Stream [56, 165]	$\cdot$			C++	AL 2.0	*N/A*
Chaos [62, 166]	$\cdot$		$\cdot$	C++	AL 2.0	*N/A*
PowerLyra [61, 167]	$\cdot$		$\cdot$	C++	AL 2.0	*N/A*
Kineograph [168, 171]	$\cdot$		$\cdot$	*Unknown*	*Unavailable*	*N/A*
Tornado [169]	$\cdot$		$\cdot$	*Unknown*	*Unavailable*	*N/A*
KickStarter [170]	$\cdot$		$\cdot$	C++	MIT	*N/A*
Pixie [173]	$\cdot$		$\cdot$	*Unknown*	*Unavailable*	*N/A*
FlowGraph [174]	$\cdot$		$\cdot$	*Unknown*	*Unavailable*	*N/A*
GPS [110, 175]	$\cdot$		$\cdot$	Java	BSD	*N/A*
GoFFish [176, 177]	$\cdot$		$\cdot$	Java	*Unknown*	*Copyright*
FBSGraph [178]	$\cdot$		$\cdot$	Unknown	*Unavailable*	*N/A*
GrapH [179, 180]	$\cdot$		$\cdot$	Java	*Unknown*	*Copyright*
Julienne [181]	$\cdot$			C++	*Unavailable*	*N/A*
GraphD [182]	$\cdot$		$\cdot$	Unknown	*Unavailable*	*N/A*
TurboGraph++ [183]	$\cdot$		$\cdot$	Unknown	*Unavailable*	*N/A*
GraphIn [184]	$\cdot$			C++	*Unavailable*	*N/A*
MapGraph [185, 186]		$\cdot$		C++	AL 2.0	*Discontinued*
CuSha [188, 189]		$\cdot$		C++	MIT	*N/A*
Gunrock [190–192]		$\cdot$		C	AL 2.0	*N/A*
Lux [193, 194]	$\cdot$	$\cdot$	$\cdot$	C++	AL 2.0	*N/A*
Frog [195, 196]		$\cdot$		C	GPL 2.0	*N/A*
Gluon [199, 200]	$\cdot$	$\cdot$		C++	*3C BSD*	*N/A*
GraphCage [205]		$\cdot$		Unknown	*Unavailable*	*N/A*
FlashGraph [207, 208]				C++	AL 2.0	*SSDs*
GraphSSD [209]				Unknown	*Unavailable*	*SSDs*

Circle $\cdot$ on the *Multi-core*, *GPU* and *Cluster* columns indicate that option is supported. *Languages* lists the programming languages the systems were written in. *License* lists the licenses of the open-source project or of the free edition of a commercial product: AL 2.0 is Apache License 2.0, CC 1.0 is Commons Clause 1.0, (GPL) v3 is GNU General Public License (GPL) v3. *Notes* covers additional information, with *Copyright* meaning that it may be illegal to reuse the source code

## Conclusion

This survey explores different aspects of the graph processing landscape and highlights vectors of research. We cover dimensions that enable the classification of graph processing systems according to the mutability of data (dynamism [112] and its modalities), the nature of the tasks (workloads where the focus may be efficient storage [129] or swift computation [210] over transient data) and how the data is associated to different computing agents (e.g., distributed via partitioning [50] to threads in a CPU, CPUs in a machine, machines in a cluster). Each of these dimensions constitutes a different branch of the study of graph processing, and herein we group their recent literature surveys and draw on their relationships. On drawing a line between graph processing systems and those that also focus on the storage, the graph databases, we found most commercial graph solutions to fall on the category of graph database. Graph databases, along the last decade, have continued to refine their efficiency in executing traversals and global graph algorithms over the graph representation stored in the database. We consider that a novel approach to extracting value from graph-based data will include the use of graph-aware data compression techniques on scalable distributed systems, potentially breaking the abstraction that these systems establish between the high-level graph data representations and the lower-level data distribution and transmission. We observe that the architecture of systems targeting graphs depend on how generic is the graph processing desired to be. Generic dataflow processing systems offer abstractions over their basic computational primitives in order to represent and process graphs, but in exchange abdicate from fine-tuning and graph-aware optimizations.

As part of our exhaustive analysis of existing contributions of different domains in the state-of-the-art of graph processing and storage, we provide direct links to source code repositories such as GitHub whenever they were available. Should the reader wish to delve into the implementation of a given contribution, a link to the contribution’s source code repository is to be found as part of the bibliography. We provide these so that other researchers and developers may look into them without need to engage in error-prone searches looking for up-to-date documentation and source-code.

This systematic analysis fosters some additional comments regarding data processing. Data is abundant, big and evolving, and paradigms such as edge computing and the evolution of the Internet-of-Things come together to reshape our relationship with data. With an increase in *smart* devices and computational capabilities becoming more ubiquitous for example in daily objects such as vehicles and smart homes, new graphs of data mapping interaction and purpose become available. This implies a continuous trend in the increasing size of data. At the same time, the dimension of dynamism (spread across the types we enumerate in this document) gains renewed importance as we move to a faster and ever-connected world. With the advent of 5G technologies and the alternative possibilities of *space internet* (among the private initiatives we count SpaceX’s Starlink, Jeff Bezos’ Blue Origin and the late Steve Jobs’ vision for an always-connected smartphone) becoming a closer reality, the temporal aspect will become even more granular.

One would not be wrong to speculate that we will have more devices which will generate data more frequently. In such a world, the graph processing dimensions we enumerate in this document will play a relevant role in building systems to handle these changing scenarios.

## Data Availability

Not applicable.
